# The Activation of Protamine 1 Using Epigenome Editing Decreases the Proliferation of Tumorigenic Cells

**DOI:** 10.3389/fgeed.2022.844904

**Published:** 2022-06-16

**Authors:** Hadjer Namous, Camila Urbano Braz, Yiding Wang, Hasan Khatib

**Affiliations:** The University of Wisconsin-Madison, Madison, WI, United States

**Keywords:** epigenome editing, PRM1, activation, proliferation, tumorigenesis, cancer

## Abstract

DNA methyltransferases (DNMT) and histone deacetylases (HDAC) inhibitors are used as cancer epigenome drugs. However, these epigenetic drugs lack targeting specificity and could risk inducing genome instability and the expression of oncogenes. Therefore, there is a need to develop new therapeutic strategies where specific cancer genes can be targeted for silencing or activation. The CRISPR/dCas9 system represents a promising, powerful therapeutic tool because of its simplicity and specificity. Protamine 1 (PRM1) is exclusively expressed in sperm and has a vital role in the tight packaging of DNA, thus inducing transcriptional silencing in sperm cells. We hypothesized that the activation of the *PRM1* gene in tumorigenic cells would lead to DNA condensation and reduce the proliferation of these cells. To test our hypothesis, we transfected human embryonic kidney cells 293T with a dCas9-P300 plasmid that adds acetyl groups to the promoter region of *PRM1* via specific gRNAs plasmids. RNA-Seq analysis of transfected cells revealed high specificity of targeted gene activation. *PRM1* expression resulted in a significant decrease in cell proliferation as measured by the BrdU ELISA assay. To confirm that the activation of *PRM1* was due to acetyl groups deposited to H3K27, a ChIP-qPCR was performed. The acetylation of the *PRM1* promoter region targeted by dCas9-p300 in transfected cells was higher than that of the control cells. Interestingly, the targeted promoter region for acetylation showed reduced DNA methylation. These findings demonstrate the efficacy of epigenome editing in activating *PRM1* in non-expressing tumorigenic cells, which could be used as a promising therapeutic strategy in cancer treatment.

## Introduction

Gene expression is orchestrated and controlled by various epigenetic regulators, such as DNA methyltransferases, histone acetyltransferases, histone methyltransferases, and other chromatin regulators involved in the addition or removal of epigenetic marks (Feinberg, 2018). Alterations of these epigenetic marks are tightly associated with tumorigenesis (Nelson et al., 2009; Verdelli et al., 2015). Additionally, the reversible nature of these epigenetics marks makes them appealing targets for cancer therapy (Wee et al., 2014). Indeed, there are several cancer drugs approved by FDA, such as 5-azacytidine and decitabine that act as DNA methyltransferase (DNMT) inhibitors or SAHA and romidepsin that act as histone deacetylase (HDAC) inhibitors (Rius and Lyko, 2012). The treatment of cancer cells with DNMT or HDAC inhibitors results in hypomethylation and hyperacetylation that can restore normal methylation and acetylation patterns, respectively, leading to the silencing or activation of genes crucial for normal cell functions (Yoo and Jones, 2006). Yang et al. (2004) reported a 1–16% and an 18–60% decrease in methylation of the repetitive elements Alu and long interspersed nucleotide elements (LINE-1), respectively, following the treatment of colon cancer cell lines with the methylation inhibitor 5-aza-2’deoxycytidine known as decitabine. Using LINE-1 as a surrogate marker for global genomic DNA methylation, Aparicio et al. (2009) found a remarkable decrease in the methylation of LINE-1 both *in vitro* and in patients with solid tumors in response to DNA methylation inhibitors. Furthermore, the treatment of colon cancer cell lines with the DNA methylation inhibitor decitabine has led to global hypomethylation, resulting in significant alterations in the expression of 70% of the genes identified in these cells (Gius et al., 2004).

Although epigenetic drugs demonstrate beneficial therapeutics for cancer, they also exhibit remarkable side effects, including a lack of targeting specificity. The lack of specificity of these inhibitors leads to genomic instability and activation of deleterious genes such as oncogenes (Yoo and Jones, 2006). Furthermore, a causal link between drug-induced epigenetic modifications and therapeutic responses to these drugs is yet to be established (Rius and Lyko, 2012). Therefore, there is a need to develop innovative approaches for targeted epigenome manipulation for gene activation or silencing specific to cancer treatment. In contrast to the existing epigenetic drugs, multiple epigenome-editing tools with targeting abilities have been developed in recent years, including TALENs, Zinc-Finger proteins, and the CRISPR dCas9 system (Jeffries, 2018). CRISPR-based systems are precise, efficient, and easy to use compared to other methods. The specificity and ease of use of the CRISPR/dCas9 system are attributed to the guide RNA (gRNA) sequences that are specific to the targeted region and the presence of the protospacer adjacent motif (PAM) sequence (Laufer and Singh, 2015). Deactivated Cas9 fused with writer or eraser proteins of epigenetic modifications, such as histone acetyltransferases and DNA methyltransferases, have proven to be efficient in activating and silencing genes *in vitro* and *in vivo* (Hilton et al., 2015; Fukushima et al., 2019; Hanzawa et al., 2020).

Recently, studies have emerged using CRISPR/dCas9 systems to activate tumor suppressor genes or to silence oncogenes. Choudhury et al. (2016) demethylated the promoter region of the *BRCA1* gene using dCas9-TET1, resulting in its expression and subsequent inhibition of HeLa cell proliferation. Song et al. (2017) demonstrated the efficiency of dCas9 fused with histone methyltransferase G9 in the down-regulation of *SPDEF*, a mucus production-related gene, in the A549 lung cancer cell line. These studies focused on targeting specific genes; however, cancer is complex, and tumorigenesis results from aberrations in the expression of multiple loci. Additionally, not every manipulated gene will inhibit cell proliferation. There is a need for an approach that focuses on suppressing abnormal cell transcriptional activity and proliferation. Protamines—proteins exclusively expressed in sperm that affect tight DNA packaging in the nucleus—present a promising tool for cancer epigenome therapy. Activation of these genes in cancer cells can lead to transcriptional silencing.

Protamines are a diverse family of short proteins (50–110 amino acids) exclusively expressed in post-meiotic male germ cells (Balhorn, 2007). They contain a central arginine-rich DNA-binding domain flanked by short peptide segments containing cysteine residues. The positively-charged arginine binds to the negatively-charged phosphate groups in DNA, while the cysteines form disulfide bridges that link the protamines together. This unique structure of protamines facilitates the packaging and condensation of sperm chromatin, preventing gene transcription (Miller et al., 2010). Transcriptional silencing in mature sperm is caused by extensive condensation of the nuclear chromatin through protamine-DNA interaction, a process called protamination (Wu and Chu, 2008; Kaur Gill-Sharma et al., 2011; Castillo et al., 2014). A few studies have reported the effect of protamines expression on chromatin packaging and the proliferation of non-germ cells. Iuso et al. (2015) demonstrated that exogenous protamine 1 (*PRM1*) expression in adult somatic cells results in higher chromatin reorganization, but no effect on cell proliferation was reported. Another study showed that exogenous expression of *PRM1* interfered with the proliferation of HeLa cells (Günther et al., 2015). Our findings demonsrate the effectivness and specificity of dCas9-p300 and two PRM1 gRNAs in activating the gene. Moreover, cells expressing PRM1 exhibited decreased cell proliferation. Also, histone acetylation correlated negatively with DNA methylation patterns at the promoter region of PRM1. Hence, the present appraoch represents a promising tool for cancer treatment.

## Materials and Methods

### gRNA Sequences Design and Plasmid Constructs

The promoter region sequence of *PRM1* was obtained from the eukaryotic promoter database (Dreos et al., 2017). The specific gRNA sequences targeting the promoter were designed with CRISPOR (Haeussler et al., 2016) using the human reference genome GRCh38/hg38. SNPs148 and Kaviar databases were used in the gRNA design to avoid mutations that could hinder the targeting efficiency of gRNAs. The sequences of the gRNAs can be found in Supplementary Table S1. The dCas9-p300 plasmid construct was obtained from Addgene (cat#61357). PRM1 gRNA expression plasmids were constructed by ligating annealed gRNA oligos using T4 ligase (NEB, United States) to psPgRNA vector (Addgene, cat# 47108).

### Cell Culture and Plasmid Transfections for PRM1 Activation

Human embryonic kidney (HEK293T) cells were purchased from Dharmacon Inc. (Horizon Discovery, United States). Cells were maintained in Gibco DMEM/F12 medium (Life Technologies, CA, United States) containing 10% (v/v) Gibco fetal bovine serum (Life technologies). Cells were incubated at 37°C in a humidified 5% (v/v) CO_2_-containing atmosphere.

For transfection experiments, cells between the third and eighth passage were seeded into 24-well plates (4 wells per treatment/control). Twenty-four hours after seeding, cells were transfected with lipofectamine 2000 (Life Technologies), 375 ng of dCas9-p300, and 125 ng of an equimolar concentration of each PRM1 gRNA expression vector. Transfections were performed in three biological replicates. The same experiments were carried out using 6-well plates where cells were transfected with 2.25 μg of dCas9-p300 and 0.75 μg of equimolar concentration of each PRM1 gRNA. We also transfected cells with dCas9-p300 alone, gRNA plasmids alone, and lipofectamine alone.

To assess the transfection efficiency in the HEK293T cell line, cells were transfected with EGFP plasmid (Addgene, cat#21320). Brightfield and EGFP fluorescent images were taken using the BIOTEK microscope (Keyence, IL, United States). A total of three images per well were taken for three separate wells. The Fiji software (Schindelin et al., 2012) was used to estimate transfection efficiency. Briefly, total cell count per well was estimated from brightfield images, while transfected cell count was estimated from EGFP fluorescence. Transfection efficiency was calculated using the following formula:

$$\%\;transfection\;=\;\frac{number\;of\;EGFP\;fluorescent\;cells}{total\;number\;of\;cells\;from\;brighfield\;image\;}\;\;$$

### *PRM1* Expression Assessment Using Real-Time Quantitative PCR (RT-qPCR)

Forty-eight hours after transfection, cells were lysed, and every four wells from 24 well plates were combined for total RNA extraction using TRIzol (Life Technologies) per the manufacturer’s instructions. When using 6-well plates, RNA was extracted from single wells. RNA quality and quantity were assessed using NanodropONE (Thermofisher Scientific, DE, United States). A total of 200 ng of purified RNA was reverse transcribed to cDNA using the iScript cDNA synthesis kit (BioRad, CA, United States). The RT-qPCR was performed using iTaq SYBR Green Supermix (BioRad). *PRM1* (target gene) and *GAPDH* (endogenous control) primers (Supplementary Table S2) were designed using the NCBI primer design tool (https://www.ncbi.nlm.nih.gov/tools/primer-blast/). RT-qPCR products were run on 2% agarose gel to confirm the presence or absence of *PRM1* mRNA amplicons in control and treated cells.

### RNA Sequencing and Differential Expression Analysis

To assess the specificity of the *PRM1* activation across the genome, we carried out RNA-Sequencing (RNA-Seq) for four transfected and four control samples. The RNA quality was assessed via Bioanalyzer 2,100 Eukaryote Total RNA Nano (Agilent Technologies, CA, United States), and all samples had a RIN >7. RNA-Seq was performed at Admera Health Biopharma (South Plainfield, NJ, United States).

For each sample, FastQC (Simon Andrews, 2010) and Trimmomatic (Bolger et al., 2010) were used to generate sequence quality reports and to trim adapter sequences and low-quality reads, respectively. Trimmed reads were aligned to the human reference genome (NCBI Homo sapiens GRCh38.p13) using STAR (Dobin et al., 2013). Read counts for each gene were estimated using the “--quantMode GeneCounts” option in STAR (Dobin et al., 2013). Only expressed genes with at least 15 counts in all four samples were considered for analysis, resulting in 13,893 genes. Gene counts were then normalized based on the trimmed mean of M-values (TMM) method (Robinson and Oshlack, 2010) using the “edgeR” R package (Robinson et al., 2010). Differential expression analysis was performed between transfected (*n* = 4) and control (*n* = 4) samples based on a negative binomial generalized linear model, including the pair of cell lines as blocking factor, using edgeR package (Robinson et al., 2010). The statistical tests were corrected for multiple testing; therefore, only genes with a false discovery rate (FDR) less than 0.10 were considered significant (Benjamini and Hochberg, 1995).

Potential off-targets of PRM1 gRNAs were predicted using CRISPOR (Haeussler et al., 2016). The resulting off-target genes were then compared to the differentially expressed genes. The specificity of gRNAs is concluded when off-targets are not present in the list of differentially expressed genes.

### Cell Proliferation Assay

To assess the effect of *PRM1* activation on cell proliferation rate, the BrdU ELISA assay was used. HEK293T cells between the fourth and eighth passages were seeded at 0.2^10^5^ cells/well in 96-well tissue culture plates. Twenty-four hours after seeding, cells were transfected with either dCas9-p300 and PRM1 gRNAs (full complex), dCas9-p300 alone, PRM1 gRNAs, or lipofectamine 2000 (see Supplementary Table S3 for transfection conditions). After 20 ± 1 h of transfection, 20 μL of BrdU reagent (Abcam, MA, United States) were added to each well containing 100 μL of DMEM/F12 medium, and cells were incubated for 22 h at 37°C with 5% CO_2_. Cells were then fixed and treated with the appropriate antibody according to manufacturer instructions. Absorbance was measured at 450 nm using a SpectraMax plate reader 384 (Molecular Devices, CA, United States). Reactions were performed in triplicate for each condition. The percent decrease in cells was calculated based on 450 nm treated/control ratio using the following formula:

$$\%\;decrease\;=1\;-\;\frac{450nm\;absorance\;in\;treated\;cells}{450\;nm\;absorbance\;in\;control\;cells\;}$$

Statistical differences between the treatment and control were assessed using a one-way *t*-test in Microsoft Excel.

### Chromatin Immunoprecipitation and ChIP-qPCR

To test whether histone acetylation deposited by dCas9-P300 at the *PRM1* promoter region leads to *PRM1* activation, chromatin immunoprecipitation assay was performed using the ChIP-IT Express Enzymatic kit (Active Motif, CA, United States). Briefly, HEK293T cells were grown in a 15-cm dish and transfected with 30 μg dCas9-p300 and 10 μg PRM1 gRNAs. After 48 h of transfection, cells were fixed with formaldehyde and harvested. Chromatin was sheared using the Active Motif Enzymatic Cocktail. A total of 20 μg of sheared chromatin were used in the immunoprecipitation reaction with 2 μg H3K27Ac antibody (Active Motif) for both control and treated cells. Pulled DNA was purified using the Active Motif DNA purification kit (Active Motif).

**FIGURE 1:**
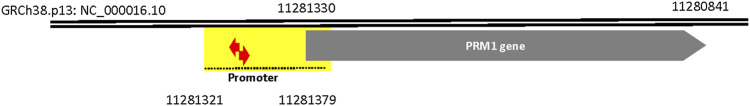
Genomic locations of PRM1 gene, promoter (dashed line), gRNAs (red arrows) and Chip region (yellow highlight). CRCh38.p13:NC_000016.10 is the chromosomal location of PRM1 gene and it promoter. The numbers on the figure are nucleotide positions.

Primers were designed to span the promoter region of *PRM1*, which is the targeted region for histone acetylation and *PRM1* gene activation (Figure 1). See Supplementary Table S2 for primer sequences. A total of 12 ng of H3K27Ac enriched gDNA from both control and treated cells were used per reaction (each sample had triplicates). gDNA was amplified using iTaq SYBR Green Supermix (BioRad). Fold change difference was calculated using the formula

$$Fold\;change\;=\;2^{-\left(PRM1\;CT\;in\;treated\;cells-PRM1\;CT\;in\;control\;cells\right)}$$

Statistical differences were obtained using a one-way *t*-test in Microsoft Excel.

### *PRM1* Promoter Region Methylation Assessment

Since histone acetylation and DNA methylation are antagonistic marks, we opted to assess DNA methylation of the promoter region targeted for acetylation. DNA was extracted from transfected and control cells using Zymo Quick-DNA Miniprep Plus Kit (Zymo Research, CA, United States). A total of 500 ng of extracted DNA was bisulfite converted using the EZ DNA Methylation-Lightning™ kit (Zymo Research). The bisulfite-converted DNA was used to amplify the *PRM1* promoter targeted for acetylation via PCR. The resulting PCR products were purified using Gel DNA recovery (Zymo Research) and ligated into the pGEM-T-easy vector (Promega, Madison, WI, United States) and transformed into JM109 competent cells (Promega). At least 20 clones per group were sequenced using Sanger sequencing. The methylation levels of single CpG sites and the overall methylation percentages were assessed using the BISMA online tool (http://services.ibc.uni-stuttgart.de/BDPC/BISMA/) (Rohde et al., 2010). Fisher’s exact test was used to test the significance of differential methylation between transfected and control cells in R software (RStudioTeam, 2020).

## Results

To activate *PRM1*, we transfected HEK293T cells with the fusion protein dCas9-p300 plasmid, which deposits acetyl groups on histones, along with gRNAs plasmids that direct the fusion protein to the promoter region of *PRM1*. Following plasmid transfection, we measured *PRM1* expression levels and assessed cell proliferation. Additionally, to understand the mechanisms of gene activation, we evaluated H3K27 acetylation and DNA methylation levels in the promoter region of *PRM1*.

### Protamine 1 Activation With dCas9-p300 and Two Specific PRM1 gRNAs

**FIGURE 2:**
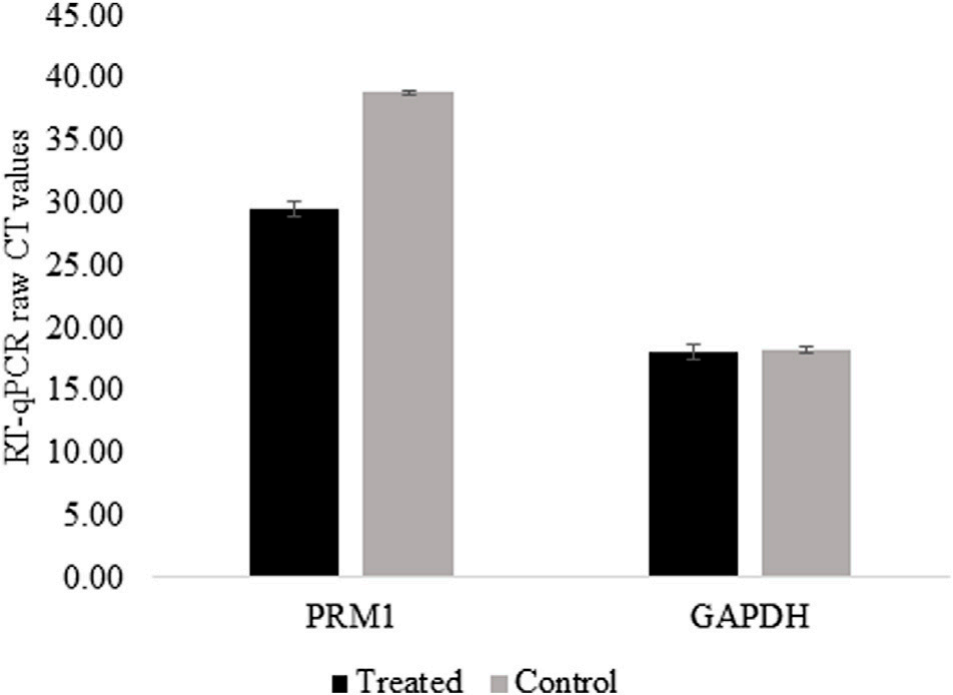
PRM1 expression in control and treated HEK293T using RT-qPCR. Raw CT values were obtained using SYBR green on BioRad CFX connect. Low CT values represent high expression of the gene. Control cells raw CTs >38 which is considered no expression. Results were obtained from three biological replicates. Error bars represent the standard error of the mean.

For *PRM1* activation, dCas9-p300 and two gRNAs specific to the *PRM1* promoter were transfected into HEK293 cells. The transfection efficiency was 31.26% based on EGFP fluorescence in transfected cells. Two days post-transfection, cells were harvested and examined for gene expression analysis. The qPCR analysis showed that non-transfected cells had no expression of *PRM1* (raw CT value > 38), whereas transfected HEK293T cells showed a raw CT value of 29.76 (Figure 2). Gel electrophoresis of the qPCR products (Supplementary Figure S1) shows that *PRM1* amplicons were present in treated cells and absent in the control cells. The *GAPDH* gene was used as the endogenous control because of its stable expression across samples, with an average CT value of 19.30 in treated cells and 19.03 in control cells. Similar to non-transfected cells, PRM1 was not expressed in cells transfected with dCas9-p300 alone, PRM1 two gRNA plasmids, and cells transfected with lipofectamine alone. These results indicate a successful activation of *PRM1* upon transfection with dCas9-p300 and two gRNAs. Additionally, we assessed the feasibility of PRM1 activation using a single gRNA; however, this approach was not sufficient, and PRM1 was not expressed in cells treated with dCas9-P300 and a single gRNA.

We performed RNA-Seq analysis on treated and non-treated HEK293T cells to determine off-targets of PRM1 gRNAs. Our findings show that predicted in-silico off-target genes were not present in the list of differentially expressed genes (Supplementary File 2). Moreover, only the *EP300* gene showed significantly higher expression in treated cells than in control cells, with a 10.77 fold difference in expression (*p* = 0.0001, FDR = 0.10). Other differentially expressed genes have fold change <2 or presented few read counts (<10). Interestingly, the expression of *PRM1* was not detected in the RNA-Seq data of all samples, although its expression was validated in RT-qPCR in the same sequenced samples.

### Cell Proliferation of Transfected HEK239T

**FIGURE 3:**
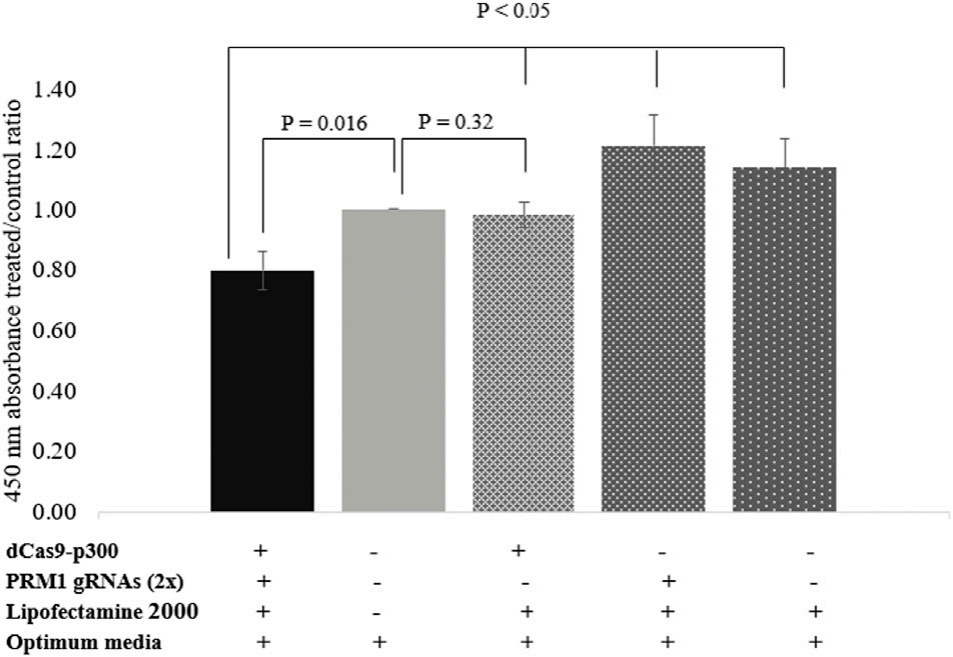
Proliferation assay using BrdU assay. Values on the *Y*-axis represent the ratio of 450 nm absorbance of treated cells to control cells. Error bars represent the standard error of the mean. Results were obtained from three biological replicates. (-) indicates absence of component in transfection complex. (+) indicates presence of component in transfection complex.

The BrdU assay was used to assess the proliferation rate of HEK293T cells following *PRM1* activation. Transfected cells expressing *PRM1* exhibited a lower absorbance at 450 nm with an average cell proliferation decrease of 20.29% compared to control cells (*p* = 0.016) (Figure 3). Moreover, treated cells showed a lower 450 nm absorbance treated/control ratio (*p* < 0.05) compared to other transfection conditions (Supplementary Table S3), indicating that *PRM1* activation is necessary to slow cell proliferation. An increased 450 nm absorbance was observed in cells treated with PRM1 gRNA plasmids plus lipofectamine 2000 (21% increase; *p* = 0.05) and only lipofectamine 2000 (14% increase; *p* = 0.124), indicating increased cell proliferation. In contrast, cells transfected with only dCas9-p300 plus lipofectamine 2000 exhibited a proliferation ratio close to that of control cells (2% decrease; *p* = 0.320).

### Histone Acetylation Enrichment in Transfected HEK293T Cells

**FIGURE 4:**
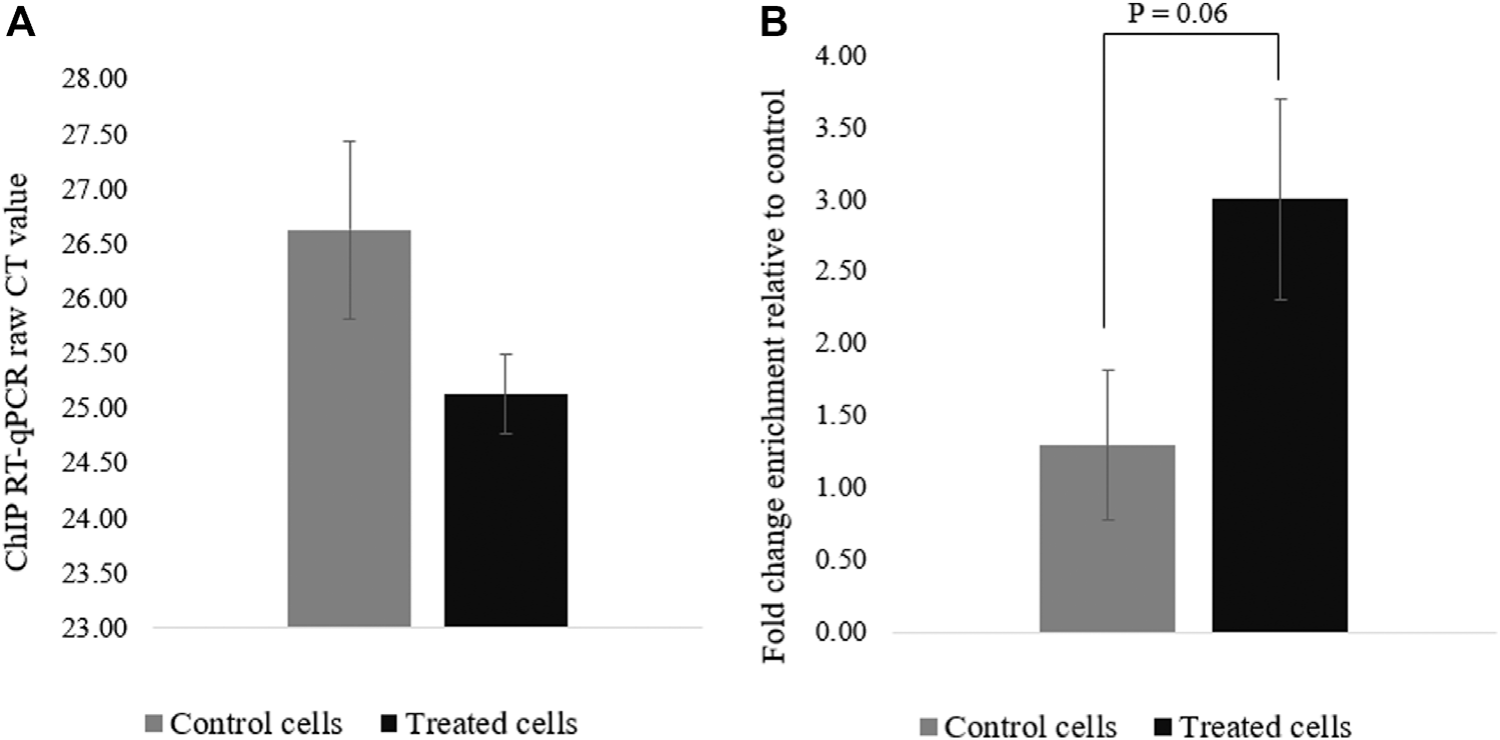
H3K27ac ChIp-qPCR CT values for control and treated cells. **(A)** Raw CT values were obtained using SYBR green**; (B)** Fold change difference (FC = 2^^−ΔCT^). Results were obtained from three biological replicates. Error bars represent the standard error of the mean.

To confirm that the deposition of acetyl groups to H3K27 by dCas9-p300 leads to *PRM1* activation, ChIP-qPCR was performed using primers specific to the region spanning the gRNAs (∼123 bp) (Figure 1). The acetylation of the *PRM1* promoter region targeted by dCas9-p300 in transfected cells was higher than that of the control cells (Figure 4). The average raw CT values of amplified H3K27ac enriched genomic DNA was 25.12 for the transfected cells compared to 26.62 for control cells (Figure 4A), indicating more H3K27Ac enrichment in treated samples where *PRM1* is expressed. The fold change enrichment was 2.82 (*p* = 0.06) (Figure 4B).

### DNA Methylation Changes in Transfected HEK293T Cells

**FIGURE 5:**
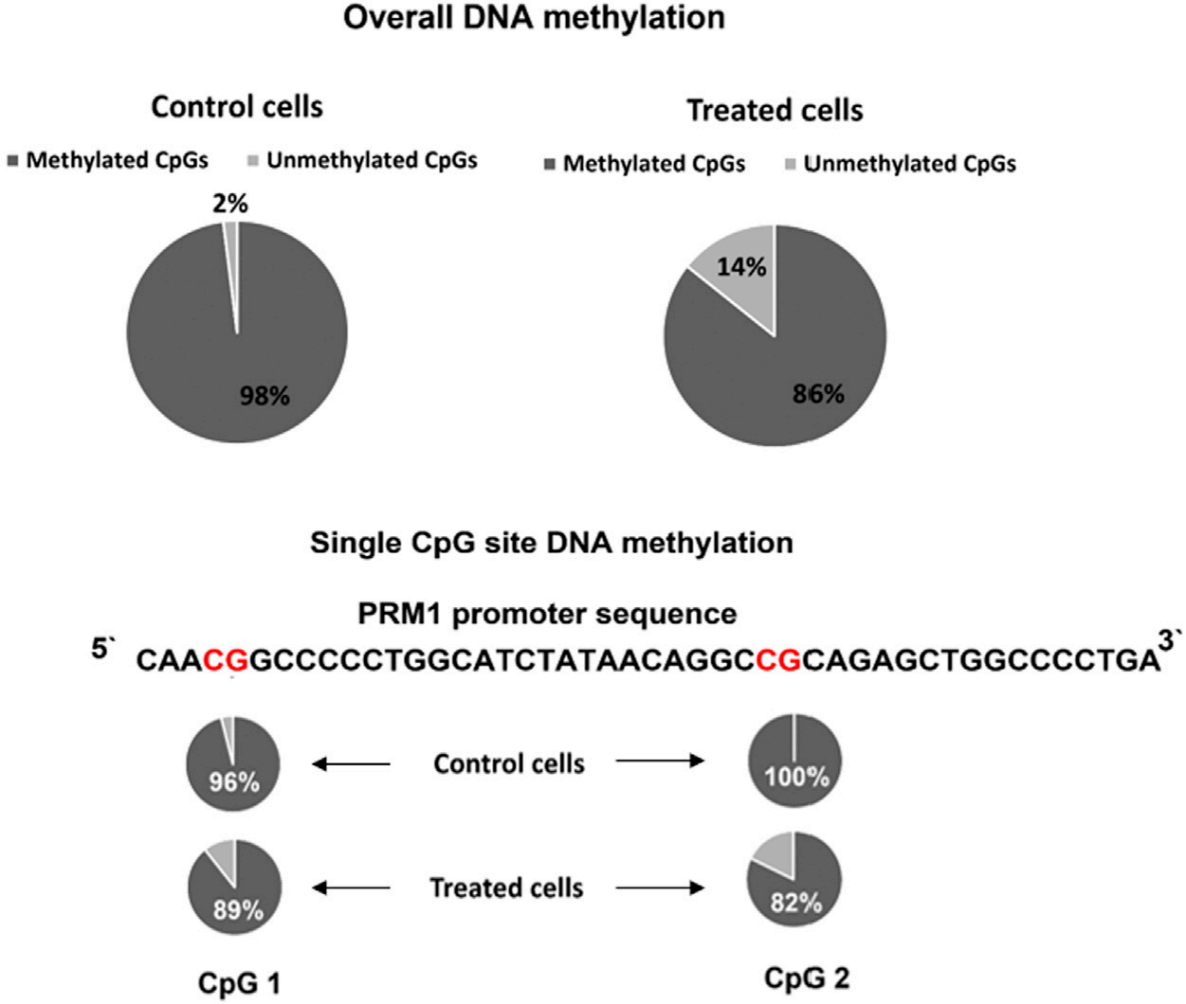
*PRM1* promoter region DNA methylation pattern in control and treated cells. Overall methylation and single CpG site methylation levels are shown. The percentage of DNA methylation per sequence was calculated using the following formula: number of methylated CpG sites/total number of successfully sequenced CpG sites. The BISMA online tool was used to assess the methylation percentage.

Histone acetylation and DNA methylation are antagonist epigenetic marks because the first activates and increases gene expression while the latter is associated with a reduced gene expression. However, the causal relationship between these epigenetic marks is not well established, and whether altering one would affect the status of the other necessitates further investigation. Thus, we assessed the DNA methylation in the region targeted for histone acetylation. The overall DNA methylation was 98% in the *PRM1* promoter region for the control cells compared to 85.71% total methylation in transfected cells (*p* = 0.034) (Figure 5). At the single CpG site resolution, CpG 1 (Figure 5) in the promoter region showed 96% methylation in control cells versus 89.28% in transfected cells (*p* = 0.61). CpG two showed 100 and 82.14% methylation in control and transfected cells, respectively (*p* = 0.053).

## Discussion

Current cancer epigenome therapeutics lack targeting specificity. Therefore, the objective of this study was to activate *PRM1* in HEK293T cells to induce transcriptional silencing in the cells and reduce their proliferation using the epigenome-editing dCas9-p300 system. Protamines confer a tight packaging of the DNA of sperm, halting transcriptional activity (Balhorn, 2007; Castillo et al., 2014); therefore, these proteins present a promising tool in the transcriptional silencing of undesired cells such as tumors. We targeted *PRM1* for activation because it is produced as a mature protein compared to *PRM2*, which requires post-translational processing to be functional. Additionally, *PRM1* is present in the sperm of all mammalian species, whereas *PRM2* has only been found in mice, hamsters, horses, some nonhuman primates, and humans (Akmal et al., 2016), which suggests that *PRM1* alone could be sufficient to induce tighter packaging of chromatin. Indeed, Iuso et al. (2015) demonstrated that *PRM1* alone induced nucleus condensation in somatic cells. Additionally, to understand the mechanism of gene activation, we evaluated the targeted region’s H3K27ac mark enrichment and DNA methylation patterns.

### dCas9-P300 Induces a Robust and Specific Activation of *PRM1*

Histone acetylation at H3K27 is tightly associated with positive regulation of gene expression (Dong and Weng, 2013; Yen et al., 2016; Zhang et al., 2020). We used dCas9-p300 fusion protein that targets H3K27 for acetylation because of its high efficiency in gene activation (Hilton et al., 2015). By targeting the promoter region of *PRM1*, we were able to activate *PRM1* in HEK293T, a tumorigenic non-germ cell line. We also successfully activated *PRM1* in the melanoma M375A cancer cell line (data not shown). Hence, our approach can be applied to different cell types.

A single gRNA in our study was insufficient to activate the targeted gene. Hilton et al. (2015) showed that using a single gRNA targeting *IL1RN* and *MYOD* promoters was enough to induce robust gene activation. In contrast, the same authors demonstrated the need for multiple gRNAs to increase the expression of the *OCT4* gene. Hence, the expression level upon activation with dCas9-p300 could be locus-specific. For *PRM1*, it is also possible that the gene’s promoter region is highly methylated and extensively condensed due to its DNA-packaging role, which necessitated using two gRNAs for successful activation.

The reported number of CRISPR/dCas9 off-target binding sites has ranged from 10 to 1,000, depending on the gRNA (Kuscu et al., 2014; Wu et al., 2014). None of the potential in-silico off-target genes was differentially expressed in our RNA-Seq analysis. Furthermore, the high expression of EP300 in treated cells was expected because of the transiently transfected P300 catalytic domain in the dCas9-p300 plasmid. Similarly, Hilton et al. (2015) reported increased expression of P300 mRNA in treated cells. These findings suggest that the PRM1 gRNAs are specific to the targeted promoter region.

The target gene PRM1 was not detected in transcriptomic analysis, probably due to its small size. Indeed, the power to detect a transcript depends on its length and abundance in the sequencing library (Oshlack and Wakefield, 2009; Sims et al., 2014). Jiang et al. (2011) demonstrated biased quantification for short transcripts and individual exons. The authors also concluded that accuracy in the observed read count values improved with read depth and RNA length. Moreover, identification of the differential expression depends on the transcript length because long transcripts generate more reads than short ones (Tarazona et al., 2011). These studies suggest that small transcripts such as *PRM1* are unlikely to be detected in the RNA-Seq data.

### Cells Expressing *PRM1* Proliferate at a Slower Rate

The decrease in the proliferation of HEK293T cells, which are considered tumorigenic (Stepanenko and Dmitrenko, 2015), indicates that *PRM1* can reduce tumor cell proliferation and presents a promising approach in cancer therapy. Similarly, Günther et al. (2015) demonstrated that transient expression of *PRM1* in HeLa cells induced a significant reduction in proliferation. In contrast, Chen et al. (2018) observed increased cell proliferation with *PRM1* overexpression in colon cancer. The authors also found that *PRM1* expression increased cell migration. Thus, investigating the role of *PRM1* in different tumor types is warranted. Although a 20% decrease seems low, it is important to note that the transfection efficiency was ∼31%. Hence, a higher decrease in estimated proliferation should be expected if transfection efficiency increases.

### The Interplay Between Histone Acetylation and DNA Methylation is a Possible Mechanism for *PRM1* Activation

The ChIP-qPCR experiments for the specific region spanning both gRNAs and the promoter region of *PRM1* showed an increase in the acetylation of H3K27 in treated cells compared to control cells. The increase in acetylation was positively correlated with the expression of *PRM1* in treated cells. These results are expected since H3K27Ac is strongly associated with active genes (Dong and Weng, 2013; Yen et al., 2016; Zhang et al., 2020). It is worth noting that transfected cells have not been sorted in this study. Thus, the relatively low transfection efficiency (∼31%) combined with a mixed population of transfected and non-transfected cells could explain the lack of significant results observed for histone acetylation enrichment. Also, enrichment of transfected cells will likely lead to increased H3K27Ac levels.

The overall DNA methylation pattern at the promoter region was reduced by 12% in treated cells upon histone acetylation enrichment. Analysis of the methylation patterns of CpG sites at the promoter region showed that the methylation level of CpG2 was lower in the treated cells than that of the control cells. These results demonstrate a negative correlation between histone acetylation and DNA methylation. It has been reported that histone methylation and deacetylation occur before DNA methylation. Wu et al. (2008) showed that low levels of H3K9 methylation resulted in poor recruitment of heterochromatin-associated protein 1 (HP1) and DNMT1 on the p16 promoter, leading to the loss of DNA methylation in the region. Other studies showed that DNA demethylase activity is directed by the state of histone acetylation (Cervoni and Szyf 2001; Cervoni et al., 2002). Moreover, D’Alessio et al. (2007) demonstrated that histone acetylation precedes but is not sufficient to trigger DNA demethylation and that RNA polymerase II (RNAP II) is necessary for the recruitment of DNA demethylase. Our attempt to activate the *PRM1* gene using dCas9-TET1 with both gRNAs was unsuccessful (data not shown). These findings suggest that *PRM1* promoter histone acetylation could be the initiation mechanism for gene expression. However, further investigation is necessary to unravel the exact mechanism and cascade of events leading to robust *PRM1* expression elongation when using dCas9-P300.

## Conclusion

This study showed that *PRM1* could be activated by targeting histone acetylation at the promoter region using dCas9-p300. Importantly, we demonstrated that protamine activation leads to a significant reduction in the proliferation of tumorigenic cells. Furthermore, the addition of acetyl groups by dCas9-P300 resulted in DNA demethylation in the promoter region, suggesting crosstalk between both epigenetic marks necessary for gene expression. Overall, *PRM1* activation in non-natively-expressing tumorigenic cells is an effective tool in reducing cell proliferation. However, this effect would likely be tumor-specific, so it is vital to assess the effect of *PRM1* activation on the cell proliferation of different cancer types. Additionally, our study focused on single cell culture in which off-tumor toxicity is not an issue. Howoever, when applying this method in a complex environment with non-tumor cells present, it is critical to use delivery tools that are tumor cell specific to prevent toxicity to normal cells.

## Data Availability

The datasets presented in this study can be found in online repositories. The names of the repository/repositories and accession number(s) can be found below: https://www.ncbi.nlm.nih.gov/, GSE188251.
