# Supplementary material for: The Activation of Protamine 1 Using Epigenome Editing Decreases the Proliferation of Tumorigenic Cells
Source: Front Genome Ed. 2022 Jun 16;4:844904. doi: 10.3389/fgeed.2022.844904 (PMC9244402; doi:10.3389/fgeed.2022.844904)
Supplement: Supplementary file 1 [file Table2.DOCX]

**Table S1.** PRM1 promoter location and gRNA sequences

| **Target** | **gRNA** **Sequence 5’ to 3’** |
| --- | --- |
| PRM1 promoter  GRCh38.p13: NC_000016.10  11281321 -11281379 | gRNA1 = TCT ATA ACA GGC CGC AGA GC |
|  | gRNA2 = GCG GCC TGT TAT AGA TGC CA |

**Table S2.** RT-qPCR, ChIP-qPCR and Bis DNA-PCR primers sequences

| **Assay** | **Target** | **Sequence 5’ to 3’** | **Size (bp)** |
| --- | --- | --- | --- |
| **RT-qPCR** | **PRM1** | Forward: TCG CAG ACG AAG GAG GCG  Reverse: CAA GAT GTG GCA AGA GGA TCT TG | 167 bp |
|  | **GAPDH** | Forward: CTG CAC CAC CAA CTG CTT  Reverse:  TTC TGG GTG GCA GTG ATG | 105 bp |
| **ChIP-qPCR** | **PRM1** | Forward: TGG CAT CTA TAA CAG GCC GC  Reverse: GAT GGG CTT GGC CTG AAT G | 123 bp |
| **Bis-DNA PCR** | **PRM1** | Forward: TTA TGG TTT GTG AGG TTT TAG TTT TTT TG  Reverse: AAA ACR ACA ACA CCC TAA TAA AAC C | 399 bp |

**Table S3.** BrDU assay transfection conditions

| **Condition** | **dCas9-p300** | **gRNAs** | **Optimen medium** | **Lipofectamine 2000** |
| --- | --- | --- | --- | --- |
| **Control** | **-** | **-** | **+** | **-** |
| **Full complex** | **+** | **+** | **+** | **+** |
| **dCas9-p300 alone** | **+** | **-** | **+** | **+** |
| **PRM1 gRNAs** | **-** | **+** | **+** | **+** |
| **Lipofectamine** | **-** | **-** | **+** | **+** |


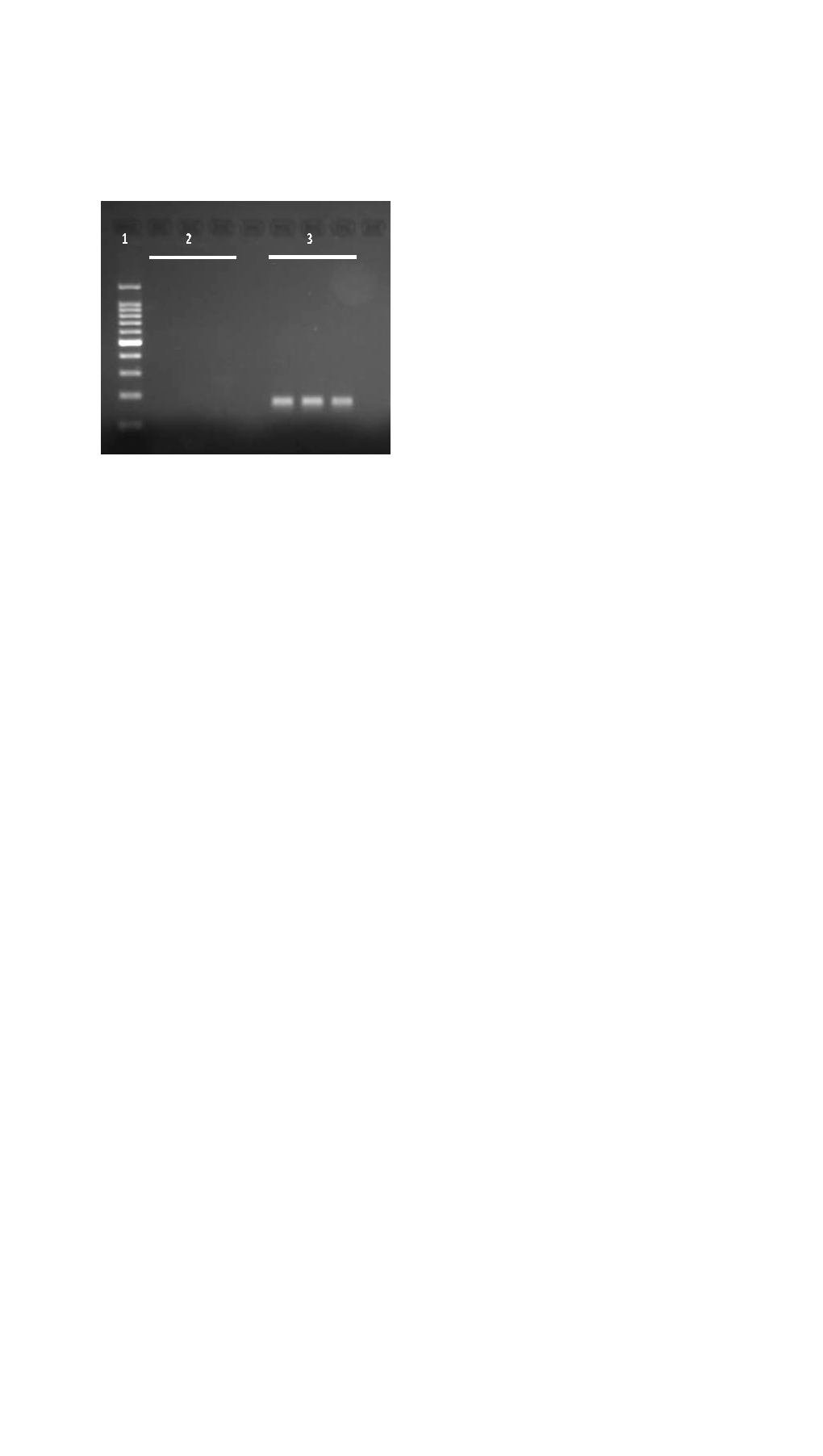


**500 bp**

400 bp

300 bp

200 bp

100 bp

**PRM1 amplicon 167bp**

**Figure 1S.** Gel (2%) image indicating expression of *PRM1* in treated cells vs absence in control cells. 1) 100 bp ladder, 2) Three wells each containing a control sample (non-treated), 3) Three wells each containing a treated sample with dCa9-p300 and PRM1 gRNAs. *PRM1* product size is 167 bp.

**Figure 2S.** PRM1 expression in control and treated M375A using RT-qPCR. Raw CT values obtained using SYBR green on BioRad CFX connect. Low CT values means high expression of the gene. For graphing purposes, we set the raw CT value of control cells to 40, however, PRM1 was not detected in control cells (raw CT values are NA).


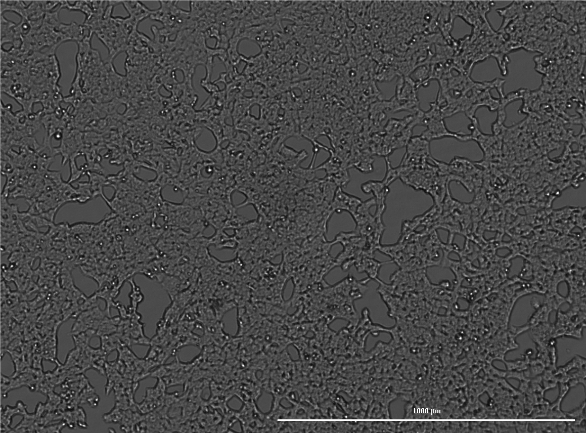


**A**


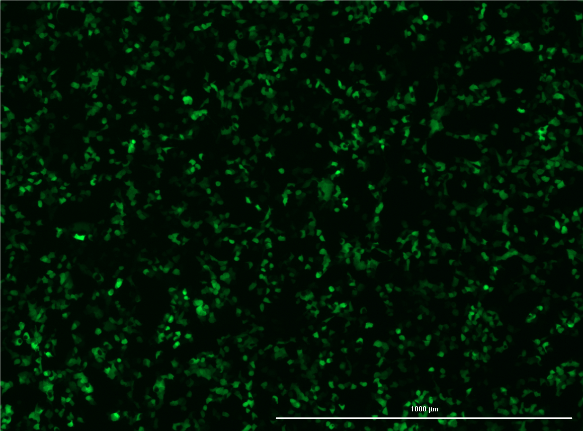


**B**

**Figure3S.** Representative Brightfield (A) and EGFP fluorescent cells (B) images in the same well
